# Public Engagement with Lung Cancer Screening Information: Topic Modeling of Lung Cancer-Related Reddit Posts

**DOI:** 10.3390/curroncol32100529

**Published:** 2025-09-23

**Authors:** Aditi Jaiswal, Samia Amin, Sayed M. S. Amin, Donghee Nicole Lee, Sungshim Lani Park, Pallav Pokhrel

**Affiliations:** 1 Department of Information and Computer Sciences, University of Hawaii at Manoa, Honolulu, HI 96822, USA; ajaiswal@hawaii.edu; 2 Department of Biology, College of Science and Engineering, Southern Arkansas University, Magnolia, AR 71753, USA; samin@saumag.edu; 3 Department of Mathematics and Computer Science, College of Science and Engineering, Southern Arkansas University, Magnolia, AR 71753, USA; samin6236@muleriders.saumag.edu; 4 Population Sciences in the Pacific Program, University of Hawaii Cancer Center, Honolulu, HI 96813, USA; nlee@cc.hawaii.edu (D.N.L.); lpark@cc.hawaii.edu (S.L.P.)

**Keywords:** lung cancer screening, public engagement, reddit, topic modeling

## Abstract

Lung cancer screening (LCS) with low-dose computed tomography is an effective strategy for early detection and improved survival. Despite its clinical benefits, public engagement with LCS topic remains unclear, particularly in the digital health communities. This study examines the thematic landscape of lung cancer-related discussions on Reddit. Using Python’s Reddit API Wrapper, we collected 109,868 posts from six lung cancer-related subreddits between January 2019 and December 2024. After preprocessing, 105,118 unique posts were analyzed using Latent Dirichlet Allocation topic modeling to identify emergent themes. Topics were qualitatively reviewed and categorized into four high-level themes: treatment, mental health, smoking, and LCS. Mental health (71.82%) and treatment (16.84%) dominated the discourse, followed by smoking (8.30%), while LCS remained underrepresented (3.04%). Despite an increase in overall engagement from 2022 onward, LCS-related posts remained sparse, with no sustained upward trend. Reddit users frequently discuss treatment and mental health concerns related to lung cancer but rarely engage with LCS as a topic, revealing a critical gap in public awareness. These findings highlight the need for targeted public health strategies to promote LCS awareness on social media platforms, leveraging the platforms’ growing role in health communication.

## Simple Summary

Lung cancer is one of the leading causes of cancer deaths, yet screening with low-dose computed tomography can save lives by detecting the disease at earlier, more treatable stages. However, many people who are eligible for screening do not take part. Social media has become an important space where patients, caregivers, and the public share experiences and seek information about health. This study examined more than 100,000 posts from lung cancer-related Reddit communities to examine public discourse. We found that discussions were dominated by treatment and mental health concerns, while lung cancer screening was rarely mentioned. This gap suggests that public awareness of screening remains low, even in spaces where people actively discuss lung cancer. Understanding these online conversations can help guide future outreach, improve health communication, and encourage greater participation in life-saving screening programs.

## 1. Introduction

Lung cancer remains the leading cause of cancer-related mortality worldwide, accounting for an estimated 1.8 million deaths annually [1,2]. Significant advances in lung cancer detection and treatment, including low-dose computed tomography (LDCT) screening [3,4,5] and targeted therapies such as tyrosine kinase inhibitors [6,7], have improved survival outcomes when the disease is identified early. With the advancement of LDCT technology, screening has emerged as an effective tool for early detection, reducing lung cancer mortality by approximately 20% in the National Lung Screening Trial (NLST) [8]. Subsequent trials and modeling studies have confirmed its mortality benefit, particularly among individuals with a history of smoking [9]. Following its initial adoption for adults aged 55–80 years with a ≥30 pack-year smoking history, annual LDCT eligibility criteria were broadened in 2021 to include adults aged 50–80 years with a ≥20 pack-year history [10,11]. Despite its proven efficacy and inclusion in international screening guidelines, LDCT remains underutilized [12], with uptake hindered by factors such as limited public awareness, fear of diagnosis, and socioeconomic barriers [13,14,15,16,17,18]. Before the 2021 criteria change, national screening uptake in the United States was even lower, just 5.8%, underscoring persistent challenges in promoting widespread adoption [19]. Understanding how the public discusses LCS is critical for identifying informational gaps regarding LCS and addressing barriers to participation.

Community-driven online spaces present unique opportunities to investigate public perceptions of LCS, identify gaps in awareness, address misconceptions, and inform targeted outreach strategies. Reddit has rapidly become a prominent platform for health-related discourse [20], particularly among individuals seeking peer support, shared experiences, and open discussions outside formal healthcare systems [21]. With over 50 million daily users [22] and topic-specific communities known as “subreddits,” Reddit facilitates open, anonymous exchanges that often reflect real-world health concerns [23,24]. Previous studies have demonstrated Reddit’s utility in monitoring public sentiment, capturing emerging health issues, and informing behavioral trends across a range of topics, including mental health [25], chronic disease [26], and cancer [27]. Its decentralized, user-driven features [23,28] offer researchers a valuable perspective for exploring grassroots conversations surrounding health conditions that carry stigma or emotional burden, such as lung cancer [29,30]. Prior analyses of online discourse reinforce this potential as studies based on Twitter and Facebook show that lung cancer conversations concentrate on awareness and treatment with little emphasis on screening [31,32]. Reddit-specific studies have examined mental health burdens and cancer-related discourse [33,34], underscoring its value for investigating patient experiences and public sentiment. Building on this foundation, our study extends the scope of online discourse analysis by systematically characterizing LCS discussions within Reddit communities and situating them alongside broader themes of treatment, smoking, and mental health.

Currently, little is known about how LCS is represented in community-driven online discussions. The present study addresses this gap by analyzing user-generated content in lung cancer-related subreddits. While our primary research question focused on the presence and nature of LCS-related discussions, we adopted a broad data collection strategy to capture all lung cancer-related conversations. This comprehensive approach was necessary because discussions of LCS may arise in diverse contexts, including those focused on treatment, symptom management, and emotional support. These communities primarily serve patients, caregivers, and survivors—groups often already diagnosed with lung cancer—where prevention and screening discourse might be less prevalent. Furthermore, given that only a small proportion of lung cancer cases are detected through screening, patient-focused communities are expected to contain relatively fewer prevention-related posts. Our research questions were: (1) How frequently is lung cancer screening discussed on Reddit? (2) What are the dominant themes in lung cancer discourse, and how do these patterns evolve over time? and (3) How do screening discussions compare to other themes such as treatment, smoking, and mental health? By analyzing all lung cancer-related discourse, we aimed not only to identify the extent of LCS discussions but also to contextualize them within the broader thematic landscape. Finally, we note that Reddit’s user base skews younger, whereas eligibility for LCS typically applies to adults aged 50 years and older. This demographic mismatch limits generalizability and may partly explain why screening is underrepresented in these online discussions.

## 2. Materials and Methods

### 2.1. Data Sources and Subreddit Selection

Data were collected from six publicly accessible Reddit communities or subreddits, e.g., r/lungcancer, r/LungCancerSupport, r/cancer, r/CancerFamilySupport, r/CancerCaregivers, and r/stopsmoking to examine lung cancer-related discussions. These subreddits were identified through a manual search using the keywords ‘lung cancer’ and ‘lung cancer screening.’ Subreddits were included if they contained a substantial volume of lung cancer-related discussions and were open to the public without access restrictions. Subreddits dedicated to highly specific cancer subtypes (e.g., non-small-cell lung cancer) were excluded to focus on broader lung cancer-related discourse and maintain thematic comparability across communities. These forums are primarily used by already-diagnosed patients and may focus on detailed treatment discussions, making them less representative of the broader discourse in which prevention and screening themes might emerge.

### 2.2. Data Collection

Original posts and their associated comment threads were extracted using the Python Reddit API Wrapper [35] over a five-year period, from 1 January 2019 to 31 December 2024. This window ensured that topic modeling incorporated temporal diversity in discourse, reflecting changes in public awareness, treatment advancements, and online engagement patterns while encompassing both pre- and post-2021 screening guideline expansions. The period was also sufficient to yield a large corpus for modeling less frequently discussed topics, such as lung cancer screening, without introducing outdated or clinically irrelevant discussions. Data collection was conducted during the early weeks of February 2025. Posts and comments not written in English were excluded. This study was exempt from ethics review board approval because it involved the analysis of publicly available, anonymized data that did not include any identifiable private or sensitive personal information [36].

### 2.3. Data Preprocessing

To ensure data quality and consistency, duplicate posts by the same user and non-textual elements such as hyperlinks and non-alphanumeric characters were removed. Text data were then processed using Python’s Natural Language Toolkit [37], following standard natural language processing techniques, including tokenization, stop word and punctuation removal, and lemmatization to extract and clean each title, body, and comment.

### 2.4. Topic Modeling and Thematic Categorization

Latent Dirichlet Allocation (LDA), a widely used unsupervised topic modeling algorithm, was applied to identify emergent patterns within the large text corpus and uncover latent themes [38]. LDA assumes that each document (i.e., Reddit post or comment) is composed of a mixture of topics, and each topic was represented by a probability distribution over words. This approach facilitated the exploration of the semantic structure of the dataset and enabled an examination of thematic evolution over time.

LDA was implemented in Python using the Gensim library [39] on the full, lemmatized corpus of Reddit posts. To determine the optimal number of topics, multiple models with varying values of number of topics or *k* (10, 15, 20, 25, 30, 35, 40) were generated and assessed through a combination of qualitative review and thematic coherence measures [40]. This iterative process revealed that a 30-topic solution best captured semantically distinct and domain-relevant themes, including topics related to LCS that were not identifiable at lower values of *k*. Each topic was characterized by its 15 to 20 highest-probability words, and the most representative posts (i.e., those with the highest topic probability) were examined to support interpretability. Topics were then manually reviewed and grouped into four higher-level themes: Treatment, LCS, Mental health and Smoking: Each post was assigned to its dominant topic (the topic with the highest posterior probability), and this topic was then mapped to one of the four themes after qualitative review. Two independent reviewers (AJ & SA) labeled the topics, achieving high interrater reliability (Cohen’s κ = 0.84) [41], with any discrepancies resolved through discussion with a senior reviewer (PP).

### 2.5. Keyword-Based Classification

In addition to LDA analysis, we implemented a keyword-based classification using curated lists of terms for each theme. These included: (1) treatment: ‘chemo’, ‘radiation’, ‘immunotherapy’, ‘surgery’, ‘oncologist’, ‘therapy’; (2) LCS: ‘low-dose CT’, ‘early detection’, ‘lung cancer screening’, ‘cancer test’, ‘biopsy’; (3) mental health: ‘stress’, ‘fear’, ‘support’, ‘depression’, ‘anxiety’, ‘cope’, ‘grief’; and (4) smoking: ‘smoking’, ‘quit smoking’, ‘cigarette’, ‘nicotine’, ‘tobacco’. Posts were flagged as relevant to a category if they contained at least one keyword from a curated list aligned with these themes. While the LDA-based method captures the broader semantic structure of discourse, including posts that may not use specific terms, the keyword-based method identifies explicit mentions of terms. This allowed us to validate the interpretation of LDA topics and to examine temporal trends in explicit language use. The relative frequency of these categories was then quantified across the dataset to estimate the prevalence and characterize patterns of each thematic area.

## 3. Results

### 3.1. Dataset Description

A total of 109,868 posts were collected across six lung cancer-related subreddits. After duplicate entries and identical content posted by the same user were removed, 105,118 unique posts were retained for analysis. Of these, 4320 were identified as original posts and 100,798 as comments, contributed by 27,170 unique users. Based on user flair information and account availability, 2 users were identified as ‘healthcare professionals (e.g., doctor, nurse)’, 27,167 were classified as ‘general’ users (whose accounts were active but did not include any healthcare-related terms), and 1 account was labeled as ‘anonymous’ (due to deleted or unavailable account identifiers). Importantly, the “anonymous” category does not represent a single individual but rather aggregates posts from deleted or unavailable accounts. In total, this category comprised 8074 posts, which all collapse under one placeholder identifier even though they originated from multiple distinct contributors.

**Figure 1:**
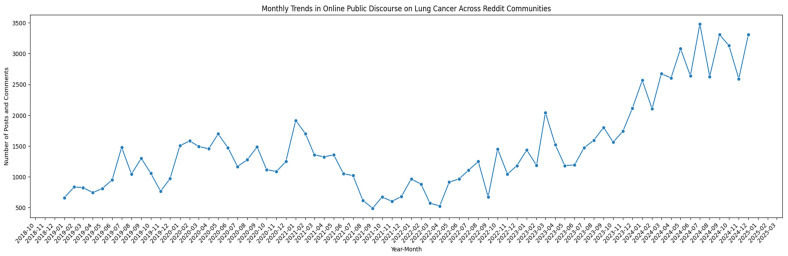
Lung cancer-related reddit discussions over time.

Figure 1 shows the total monthly volume of Reddit posts and comments related to lung cancer across the subreddits analyzed in the present study. Both original posts and their associated comment threads were included in these data. Although fluctuations in the volume of posts and comments were observed throughout the period, the overall trend showed a rise in engagement over time, especially in 2023 and 2024.

### 3.2. Keyword-Based Thematic Distribution

**Figure 2:**
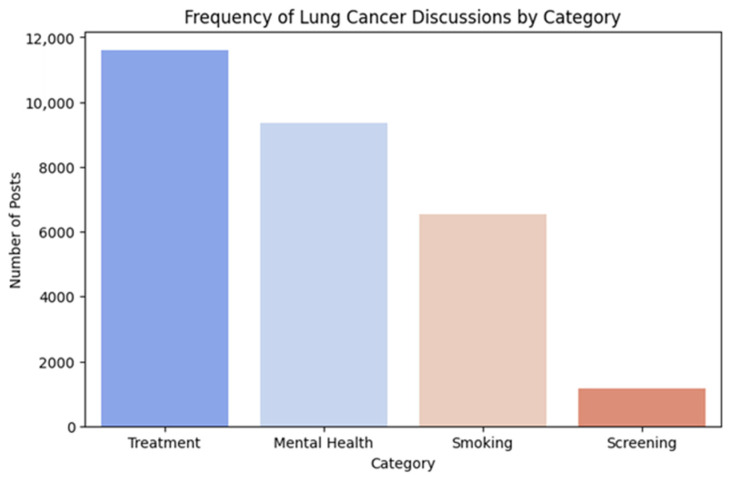
Frequency of lung cancer-related reddit discussions by category.

Using curated keyword lists, posts were categorized into four themes: treatment, mental health, smoking and LCS. Figure 2 shows the overall frequency of posts across these categories.

Among the 105,118 unique posts analyzed, ‘treatment’-related posts were most prevalent, with 11,600 posts (11.03%), followed by ‘mental health’ with 9360 posts (8.90%), and ‘smoking’ with 6539 posts (6.22%). However, ‘LCS’-related posts were markedly underrepresented, with a total of 1176 posts (1.12%). It is important to note that these counts do not sum to the total corpus because the keyword lists were selective and non-exhaustive. Posts using alternative terminology or informal phrasing may not have been captured, so the estimates should be viewed as lower bounds of thematic prevalence rather than definitive totals. In addition, many posts spanned multiple themes (e.g., users describing anxiety before chemotherapy or referencing smoking cessation alongside screening results). Because the keyword tallies are based on explicit mentions rather than contextual overlap, these multi-faceted narratives may be oversimplified, providing a partial but useful view of thematic emphasis within the broader discourse. Table 1 lists the examples of posts on topics within each category.

### 3.3. Keyword-Based Temporal Trends

**Figure 3:**
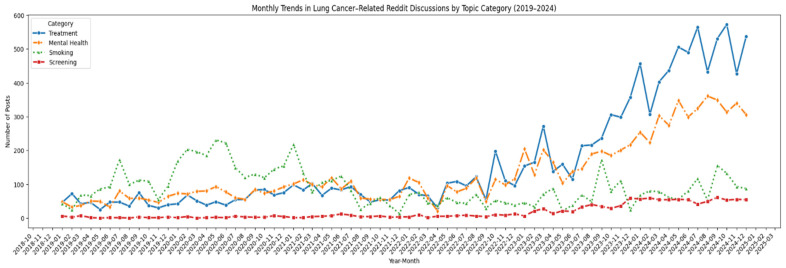
Trends in lung cancer reddit discussions related to treatment, lung cancer screening, mental health, and smoking.

Figure 3 shows the monthly trends in keyword-based counts across the four categories from January 2019 to December 2024. An upward trend was observed in ‘treatment’- and ‘mental health’-related discussions, particularly from 2022 onwards. ‘Smoking’-related discussions fluctuated without a consistent pattern. ‘LCS’ remained the least discussed topic throughout the period, with only a modest increase in late 2023, which did not lead to sustained attention over time.

### 3.4. Topic Modeling Results Using LDA

For the LDA analysis, model performance was assessed using coherence scores across values of *k* ranging from 10 to 40. Coherence values decreased from 0.5665 at *k* = 10 to 0.4463 at *k* = 40. A 30-topic solution (coherence = 0.4747) was selected as it offered the best trade-off between interpretability and detail, including clearer separation of screening-related topics. Table 2 summarizes the results of the topic modeling analysis, with representative topics listed under the four manually assigned higher-level themes. Each topic was accompanied by its word distribution (top contributing keywords), the total number of associated posts, and the percentage of the full dataset the respective posts represented. Among the analyzed posts, the majority (71.82%) were assigned to ‘mental health,’ followed by ‘treatment’ (16.84%), ‘smoking’ (8.30%), and ‘LCS’ (3.04%).

## 4. Discussion

The present data revealed that treatment and mental health-related discussions were most prevalent among Reddit users who participated in discussions on lung cancer, while posts on LCS remained notably underrepresented. Despite growing public health efforts to promote early detection through low-dose CT screening worldwide [5], only 1.12% of the posts explicitly addressed LCS, and only 3% of posts were thematically linked to LCS in the topic modeling analysis. These findings highlight a significant gap in public awareness and engagement on social media regarding LCS, a critical public health strategy for early detection and improved outcomes.

The volume of Reddit discussions related to lung cancer increased between 2019 and 2024; particularly between 2023 and 2024, when the increase was more drastic. This surge may reflect multiple converging factors, including an increased use of social media platforms such as Reddit for health-related conversations and peer support [42,43], as well as potential downstream effects of the 2021 expansion of LDCT screening eligibility criteria, which may have heightened public awareness and prompted greater online engagement with lung cancer-related topics. Prior research indicates that Reddit has become an important source of community-based health information, particularly for topics that may be considered sensitive or stigmatizing [44,45,46]. The fluctuations seen in earlier years may reflect episodic events or news cycles that temporarily heightened interest, while the sustained rise in later years points to a broader cultural shift toward using Reddit as a platform for health discourse. Prior studies have highlighted that Reddit users often seek both informational and emotional support from other users on the platform, especially when access to professional health services is limited or delayed [21,27,47,48]. The implication of this finding is significant for public health professionals, as it underscores the potential of Reddit as a tool for real-time health surveillance, dissemination of accurate health information, and engagement with at-risk populations. Furthermore, understanding the dynamics of these discussions can inform the development of targeted interventions and communication strategies that resonate with online communities. Future research should explore the content and sentiment of these discussions in more detail, assess the credibility of shared information, and evaluate how engagement with Reddit affects health-related decision-making and behaviors.

From 2022 onwards, there was a notable increase in public engagement with topics related to lung cancer treatment and mental health experiences of individuals directly affected by lung cancer such as patients, survivors and care-providers. This pattern is consistent with prior cancer-related research, which has found that people are increasingly using social media platforms for sharing treatment experiences, providing or seeking emotional support, and navigating through a complex care decision-making process [49,50]. The sharp rise in mental health discussions may reflect increasing recognition of the psychological burden faced by cancer patients and survivors, as well as greater openness to discussing mental health challenges in digital spaces. Recent studies highlight higher rates of anxiety and depression among lung cancer patients compared with patients with other types of cancer [51].

The surge in treatment-related content may be attributed to recent advancements in immunotherapies, targeted therapy for actionable mutations and patient-driven demand for information on emerging lung cancer therapies. In fact, the current analysis revealed that for public conversation on lung cancer, treatment-related content was dominant followed by mental health, smoking, and screening. Prior research indicates that patients and caregivers often turn to online forums to seek and share information about treatment options, side effects, and therapeutic experiences [52]. In contrast, the current data shows that the screening-related discussions remained low throughout the study period, which may indicate potential lack of awareness regarding LCS in the public discourse. This study underscores the need to enhance digital health literacy and target awareness campaigns more effectively on digital platforms, consistent with prior research showing that social media can raise knowledge of LCS [53], digital health literacy is critical for navigating online health information [54], and social media is widely used for health purposes including awareness and support [42]. Furthermore, despite the well-established link between tobacco use and lung cancer, the current data showed comparatively fewer discussion related to tobacco smoking or smoking cessation. This may be an indication of the possibility of the lack of awareness or stigmatization in the social media users regarding certain smokers being eligible for LCS. Clearly, more research is needed to test this proposition.

A major strength of the present analysis is that it is based on over 105,118 unique Reddit posts across six lung cancer-focused communities over a five-year period. Thus, the analysis is likely to have offered a comprehensive, real-world view of public discourse. Using LDA allowed for the discovery of latent themes without imposing researcher bias, capturing organically emerging conversations. Posts were categorized into four key domains (treatment, mental health, smoking, and screening), enabling structured comparison of thematic analysis. Monthly post trends were examined from 2019 to 2024, revealing dynamic shifts in public engagement with lung cancer topics over time.

This study has several limitations. First, the data were limited to publicly accessible Reddit communities and may not reflect broader population-level discourse or perspectives outside of Reddit users. The social media platforms user base skew younger (average age = 23 years) and more technologically engaged population [55,56] while the median age of lung cancer diagnosis in the United States is approximately 70 years [57], thus limiting generalizability of this study. This demographic mismatch may influence the types of concerns voiced and the level of clinical awareness while limiting the representativeness of Reddit discourse for the broader lung cancer screening-eligible population. Second, user anonymity and the lack of demographic metadata restrict our ability to stratify discussions by age, gender, socioeconomic status, or geographic region—factors known to influence health behaviors and screening feasibility. Most participants remained anonymous or unverified, limiting our ability to differentiate between patients, caregivers, and professionals, complicating interpretation of themes. Third, while LDA is a widely used topic modeling approach, it is inherently limited in its ability to capture complex semantics and contextual nuance and topic interpretability is influenced by parameter selection (e.g., number of topics). Our thematic categorization, though carefully reviewed, required manual labeling, which introduces subjectivity and may limit reproducibility. Additionally, individual posts may span multiple themes, yet our framework assigns each post to a single dominant topic, potentially oversimplifying the thematic landscape.

Fourth, although our thematic prevalence estimates were derived from LDA, we also conducted sensitivity checks using the keyword-based classification of posts into four thematic categories that may have introduced bias. Some keywords may not accurately capture the intended theme in all contexts, and posts containing relevant content but lacking specific keywords may have been missed and people may use diverse or informal expressions when discussing topics such as lung cancer screening. As a result, it is not feasible to capture every possible phrasing, and some relevant posts may not have been identified. Fifth, while smoking-related discourse was captured under the “Smoking” theme, we categorized smoking separately from screening to maintain analytic clarity. This means our analysis did not directly examine the intersection of tobacco cessation and LCS, which may be an important area for future investigation. Sixth, reduced access to routine screening during the pandemic likely contributed to the relative scarcity of the screening-related discussions prior to 2022. Seventh, stigma associated with smoking and the eligibility criteria for lung cancer screening (which prioritize individuals with a smoking history) may have influenced who chose to participate in Reddit discussions and how screening was discussed. Because our dataset lacked reliable metadata on user smoking status, we could not directly evaluate this effect, but it remains an important contextual factor to consider. Finally, as analyses were confined to Reddit, findings may not generalize to broader social media platforms or offline health conversations.

Despite the limitations, this research sheds light on a major gap in public engagement with LCS in digital forums, despite its proven value in early detection and mortality reduction. Future work could build on this study in several important ways. First, expanding analyses to platforms with older user bases or integrating survey data from screening-eligible adults could help address the demographic mismatch between Reddit users and the population targeted for lung cancer screening. Second, incorporating datasets with demographic or behavioral metadata (e.g., smoking history, socioeconomic status, geographic region) would enable a more nuanced understanding of how these factors shape online discourse. Third, methodological advances such as multi-label classification, hierarchical topic modeling, or contextual embedding models could capture posts that span multiple themes and preserve greater semantic nuance beyond dominant-topic assignment. Fourth, future work could extend this analysis to subtype-specific forums (e.g., non-small-cell lung cancer), which may provide additional insights into how screening is discussed within patient communities already engaged in clinical care. Fifth, future studies could more explicitly investigate the intersection between smoking cessation discourse and screening awareness, given that tobacco use remains central to eligibility criteria and may influence willingness to engage with screening. Finally, expanding analyses beyond Reddit, to include subtype-specific patient communities or other social media platforms, could provide complementary perspectives on how screening is discussed across more specialized and diverse digital spaces.

The findings have practical implications for public health professionals, digital strategists, and cancer advocacy organizations. By highlighting the underrepresentation of LCS as a discussion topic within Reddit communities, this study underscores the need to enhance digital health literacy and target awareness campaigns more effectively on digital platforms. In particular, outreach efforts could benefit from approaches that make screening content more visible and engaging within online communities. Moreover, the study positions Reddit as a valuable resource for health surveillance, offering timely insight into patient concerns and gaps in knowledge that may not be captured through traditional surveys or clinical interactions.

## 5. Conclusions

In summary, this study reveals a striking underrepresentation of LCS in Reddit discussions, despite increasing use of the platform for cancer-related support and information. Mental health among lung cancer patients, survivors, and caregivers, as well as lung cancer treatment, dominate the discourse. The limited visibility of LCS in these conversations highlights an important gap in the salience of screening within online communities, even among individuals actively discussing lung cancer. While these results should not be interpreted as a direct measure of population-level awareness, given that Reddit users skew younger than the screening-eligible population (aged 50 years and older), it underscores the need for targeted digital outreach and health communication strategies. Addressing this gap and strengthening the presence of screening-related content online, for example through survivor advocacy to share personal narratives, structured engagement initiatives within Reddit communities (e.g., Ask-Me-Anything sessions with clinicians or pinned educational posts), or integration into broader cancer discussions, could improve the visibility of LCS and ultimately improve early detection efforts.

## Figures and Tables

**Table 1:** Example posts for each category.

Category	Example Posts
Treatment	1. It took me about 6/7 months after treatment ended to work up to walking a mile and 10 months to build back to walking 2 miles. I was 58 at that time……chemo regimen was much more difficult then.
	2. Not sure if she will qualify since they usually do the chemo and immunotherapy first then surgery.
	3. I just got my latest scan results back on Monday……Immunotherapy is working……I’m so happy……I’m continuing with treatment and see what the next round of scans says in July!
Mental health	1. This is a brutal disease and it breaks my heart that so many other people have to suffer through it. I’m so grateful to have this supportive group, because no one should have to go through this on their own.
	2. Friends and family try their hardest but they don’t understand nor do I expect them to……I don’t want people to see me as just a sick girl. I don’t want all the memories I have with friends to be forever overwhelmed by them seeing me on my death bed.
	3. One of my close friends lost his dad to cancer, the same kind that my dad is currently suffering from. My friend told me that what helped him was to not rush the process.
Smoking	1. In my experience the feeling does go away. It will take some time, but cravings will stop. Sometimes when I’m out and I smell a freshly lit cigarette I get a momentary craving, but that’s easily squashed with resolve and will power.
	2. I still think about smoking most days, usually in the evening when I used to hang out in the garage and have a cig and a beer. I miss that quite a bit, and don’t want to be chained to that longing forever.
	3. I should stop now before the addiction gets worse……dealing with withdrawals when I quit, I would close my eyes for a moment, do a deep breathing exercise, and tell myself "It’s going to pass whether you have a cigarette or not. Just ride it out.
Screening	1. I met with a pulmonologist a few weeks after I was initial diagnosed. He didn’t do much but say that we needed a biopsy. I got the biopsy, which confirmed it was cancer, and oncology took over.
	2. Feeling very terrified right now. My mother had a CT scan which showed she has multiple lung nodules, and she has never been a smoker. She has asthma and uses an inhaler, and has had several spouts of coughing which has now gone away, and she has never coughed up blood before……I don’t know what the lung nodules mean or whether they mean cancer, or how common finding a non-benign nodule is.

**Table 2:** Summary of categories, relevant words, posts numbers and percentage.

Category	Relevant Words	Posts Numbers	Percentage
Treatment	effects, radiation, terminal, insurance, recovery, remove, nausea, therapy, palliative	17,700	16.84%
Mental health	advice, helpful, care, support, family, peace, panic, hugs, anxiety	75,497	71.82%
Smoking	quit, smoke, vaping, uncomfortable, progress, patches, withdrawal	8724	8.30%
Screening	symptoms, early, doctor, risk, rare, scan, biopsy, test	3197	3.04%

## Data Availability

Data are available upon request to the corresponding author.
